# Challenges and potential improvements in the Accreditation Standards of the Korean Institute of Medical Education and Evaluation 2019 (ASK2019) derived through meta-evaluation: a cross-sectional study

**DOI:** 10.3352/jeehp.2024.21.8

**Published:** 2024-04-02

**Authors:** Yoonjung Lee, Min-jung Lee, Junmoo Ahn, Chungwon Ha, Ye Ji Kang, Cheol Woong Jung, Dong-Mi Yoo, Jihye Yu, Seung-Hee Lee

**Affiliations:** 1 Department of Medical Education, Seoul National University College of Medicine, Seoul, Korea; 2 Department of Medical Education, Hanyang University College of Medicine, Seoul, Korea; 3 Department of Surgery, Korea University College of Medicine, Seoul, Korea; 4 Departments of Medical Education, College of Medicine, The Catholic University of Korea, Seoul, Korea; 5 Department of Medical Education, Ajou University School of Medicine, Seoul, Korea; Hallym University, Korea

**Keywords:** Meta-evaluation, Medical education accreditation, Quality improvement, Cross-sectional study

## Abstract

**Purpose:**

This study aimed to identify challenges and potential improvements in Korea's medical education accreditation process according to the Accreditation Standards of the Korean Institute of Medical Education and Evaluation 2019 (ASK2019). Meta-evaluation was conducted to survey the experiences and perceptions of stakeholders, including self-assessment committee members, site visit committee members, administrative staff, and medical school professors.

**Methods:**

A cross-sectional study was conducted using surveys sent to 40 medical schools. The 332 participants included self-assessment committee members, site visit team members, administrative staff, and medical school professors. The t-test, one-way analysis of variance and the chi-square test were used to analyze and compare opinions on medical education accreditation between the categories of participants.

**Results:**

Site visit committee members placed greater importance on the necessity of accreditation than faculty members. A shared positive view on accreditation’s role in improving educational quality was seen among self-evaluation committee members and professors. Administrative staff highly regarded the Korean Institute of Medical Education and Evaluation’s reliability and objectivity, unlike the self-evaluation committee members. Site visit committee members positively perceived the clarity of accreditation standards, differing from self-assessment committee members. Administrative staff were most optimistic about implementing standards. However, the accreditation process encountered challenges, especially in duplicating content and preparing self-evaluation reports. Finally, perceptions regarding the accuracy of final site visit reports varied significantly between the self-evaluation committee members and the site visit committee members.

**Conclusion:**

This study revealed diverse views on medical education accreditation, highlighting the need for improved communication, expectation alignment, and stakeholder collaboration to refine the accreditation process and quality.

## Graphical abstract

[Fig f1-jeehp-21-08]

## Introduction

### Background

Advancements in the medical field directly impact national health levels. Within this framework, the caliber of medical school education is widely acknowledged as a crucial element in global health policies. The production of well-trained medical professionals is vital for bolstering a country’s health infrastructure and enhancing the provision of medical services. In light of this significant duty, Korea has been actively standardizing and enhancing medical school education by implementing accreditation standards since 2000 [1]. The Korean Institute of Medical Education and Evaluation (KIMEE) is at the forefront, serving as the principal body responsible for managing the evaluation and accreditation of medical education in Korea [2]. The Accreditation Standards of KIMEE 2019 (ASK2019) were developed to improve the medical school education standard and update the educational content to align with international benchmarks, notably the World Federation for Medical Education Global Standards for Quality Improvement [3]. There are 9 evaluation domains, 36 sub-areas, 92 basic standards, and 51 high-quality standards in ASK 2019. Compared to previous evaluation standards, although the total number of items has mainly remained consistent, there has been a noticeable expansion in evaluation areas and divisions, resulting in a more refined and comprehensive assessment framework [4]. The accreditation and evaluation process for medical schools in Korea includes a self-study by the school, collection, and review of self-study and student reports, an on-site visit, and a final decision on accreditation status. While numerous studies have indicated that the evaluation and accreditation system has positively influenced various aspects, such as curriculum development and the enhancement of student performance [5], a comprehensive analysis and understanding of these systems’ specific operational processes and outcomes still need to be improved.

Meta-evaluation has been an effective tool for assessing the accreditation process, as it critically examines the evaluation process itself [6]. In Korea, meta-evaluation methods have been used to assess university certification evaluations across various disciplines [7,8]. The KIMEE has conducted a meta-evaluation from 2011 until the second cycle of the third round of evaluation and certification of Korean medical schools.

Past meta-evaluations proved that the standards used to evaluate medical school accreditation were effective in promoting quality improvement and accountability in healthcare. However, they needed to catch up in capturing certain qualitative aspects that could not be measured with quantitative methods, indicating a notable gap in assessment. In light of this, a comprehensive meta-evaluation of the newly implemented ASK2019 was essential.

### Objectives

This study aimed to perform a comprehensive meta-evaluation of medical school accreditation and evaluation based on ASK2019 to identify areas for improvement and potential future directions. To achieve this goal, the study systematically collected and analyzed the insights and perceptions of a diverse group of stakeholders. This group included members of the self-assessment committee, the local visiting committee, administrative staff, and medical professors, all within the operational framework of ASK2019.

## Methods

### Ethics statement

The Hanyang University Institutional Review Board (IRB no., HYUIRB 202307-011) approved this study, classifying it as low-risk and exempting it from the requirement for written consent because it utilizes anonymous email surveys that participants returned after giving consent.

### Study design

This cross-sectional, national-level study involving stakeholders from 40 medical schools was conducted using an online survey.

### Setting

From September 2022 to February 2023, over 5 months, letters requesting cooperation were sent to 40 medical schools in Korea, facilitated by the coordination and recommendation of the KIMEE. To collect survey responses, the KIMEE sent these letters to medical professors and administrative staff at the 40 medical schools. Surveys were also distributed to the site visit committee members and the self-evaluation committee via the email addresses registered with the KIMEE, ensuring a thorough data collection process. The research team acquired and analyzed anonymized data from the KIMEE.

### Participants

The survey participants comprised 396 individuals, including professors from 40 national medical schools, members of self-evaluation committees with experience in medical school accreditation processes, and site visit committee members from the KIMEE who have participated in the evaluation and accreditation of medical schools. Additionally, administrative staff and faculty members from each medical school were part of the cohort. Participation in the survey was voluntary.

#### Variables

The variables were derived from Daniel Stufflebeam’s CIPP (Context, Input, Process, Product) model [9] and were finalized after consulting with experts on the validity of each item. Context evaluation is the Overall Perception of Accreditation, which corresponds to understanding the necessity and contribution of accreditation, alongside assessing the positive outcomes and the reliability, fairness, and objectivity of KIMEE. Input evaluation involves assessing the resources, strategies, and plans before the accreditation process begins. This procedure aligns with evaluating the appropriateness and clarity of accreditation standards and the feasibility of implementing these standards. In-process evaluation evaluates the procedures and methods used during the accreditation process, including addressing challenges in preparing self-evaluation study reports. Finally, product evaluation looks at the outcomes of the accreditation process, including the appropriateness of resources allocated to site visits, perceptions of the site visit committee members, and the accuracy of site visit evaluation results.

### Data sources/measurement

The research team developed the survey items collaboratively and subsequently refined through iterative discussions with the KIMEE’s Quality Improvement Committee. Experts experienced in accreditation to ensure the survey’s validity and reliability (Supplement 1). The survey encompassed various aspects of accreditation, such as its necessity, contribution to medical education, and perceptions of the KIMEE’s credibility, fairness, and objectivity. Additionally, it assessed the clarity and applicability of the accreditation standards and procedures. Responses were collected using a 6-point Likert scale for all items, and multiple-choice questions were employed to assess the positive outcomes of accreditation and the challenges encountered in preparing self-evaluation study reports. Context, input, process, and product evaluation criteria for the ASK2019 program are available in Supplement 2.

### Bias

To minimize selection bias, we conducted surveys with one representative from each group: the self-evaluation study committee, the site visit committee, administrative staff, and faculty members from all 40 medical schools in South Korea.

### Study size

Using G*Power (Heinrich-Heine-Universität Düsseldorf) [10] and assuming a medium effect size (F=0.25) [11] at a 0.05 significance level, at least 280 participants were needed for one-way analysis of variance (ANOVA) to ensure a statistical power of 0.95. Considering potential dropouts, this study recruited 300 participants.

### Statistical methods

Data analysis was conducted using IBM SPSS ver. 25.0 (IBM Corp.). The collected data comprised basic statistics, including mean and standard deviation. The t-test, one-way ANOVA, and the chi-square test were utilized to compare perceptions of medical education accreditation among 4 distinct groups: self-evaluation study committee members, site visit evaluators, general medical faculty, and medical school administrative staff.

## Results

### Participants

After excluding missing data, the study included 332 participants. This cohort consisted of 172 self-evaluation study committee members, 28 site visit evaluators, 69 general professors from medical schools, and 64 administrative staff members from medical schools (Table 1).

### Context evaluation: overall perception of accreditation response data of the survey are available at Dataset 1.

#### Necessity of accreditation

Statistically significant differences were observed in the perceived necessity of accreditation among the 4 groups (Table 2). The most significant difference was noted between the site visit committee members and medical school faculty members.

#### Contribution to accreditation and evaluation

As shown in Table 3, there was no significant difference in the responses between the self-evaluation study committee members and medical school professors regarding how much the evaluation and accreditation process contributed to improving the quality of medical education at their respective medical schools. Both groups exhibited predominantly positive attitudes.

#### Positive outcomes of accreditation

No significant differences were observed among the groups regarding the positive outcomes of accreditation (Table 4). Members of the self-evaluation study committee and administrative staff predominantly perceived the accreditation process as beneficial, with improvements in areas previously identified as deficient during the self-evaluation study being the most frequent indication.

#### Reliability, fairness, and objectivity of the KIMEE

Significant differences were observed in how members of the self-evaluation study committee and administrative staff perceived the reliability, fairness, and objectivity of the KIMEE, as shown in Table 5. Across all 3 aspects, administrative staff held a more favorable opinion of the KIMEE than the self-evaluation study committee members.

### Input evaluation: perception of accreditation standards

#### Appropriateness and clarity of accreditation standards

Significant differences were observed between the self-evaluation study committee members and the site visit committee members regarding opinions on accreditation standards. These differences pertained to the adequacy of basic criteria, excellence criteria, and the clarity of the standards (Table 6). Notably, site visit evaluators had more positive perceptions than self-assessment committee members for all 3 items.

#### Feasibility of implementing accreditation standards

Regarding the feasibility of implementing evaluation and accreditation standards in medical education, notable differences were observed in the average responses among self-evaluation study committee members, site visit committee members, and administrative staff (Table 7). Specifically, the disparity between the perceptions of administrative staff and self-evaluation study committee members was the most marked for perceived feasibility.

### Process evaluation: perceptions of the accreditation process

#### Appropriateness of medical education accreditation procedures and methods

Significant differences were observed in the average perceived suitability of the current accreditation processes between self-evaluation study committee members and administrative staff, except for the interim evaluation report (Table 8). Administrative staff held a more favorable opinion of the appropriateness of medical education accreditation procedures and methods than self-evaluation study committee members.

#### Challenges encountered in the preparation of self-evaluation study reports

The distribution of challenges faced while preparing self-evaluation study reports varied among different stakeholder groups in medical schools (Table 9). Self-evaluation study committee members reported that the most significant difficulty was a need for a clearer understanding of accreditation standards (23.8%), while administrative staff primarily found duplication of content (35%) to be the most challenging aspect.

### Product evaluation: perceptions of site visit evaluations

#### Appropriateness of resources allocated to site visits

Site visit committee members had more positive perceptions than self-evaluation study committee members regarding the appropriateness of resource allocation for site visits (Table 10). However, there was no significant difference between the 2 groups in terms of cost appropriateness.

#### Perceptions of the site visit committee members

There was no significant difference between the self-evaluation study committee members and the site visit committee members regarding the professionalism of the site visit committee (Table 11).

#### Perceptions of site visit evaluation results

Regarding the accuracy of the final evaluation report, a significant difference was observed between the self-evaluation study committee members and the site visit committee members (Table 12). Site visit committee members had more positive perceptions than self-evaluation study committee members.

## Discussion

### Key results

This study aimed to explore the diverse perspectives of key stakeholders engaged in medical education accreditation in South Korea. These stakeholders included members of the self-evaluation study committee, site visit committee members, administrative staff, and medical professors. During the meta-evaluation process, a cross-sectional survey was administered. The findings revealed that members of the self-evaluation study committee and medical professors generally held positive views regarding the role of accreditation in enhancing medical education.

### Interpretation/comparison with previous studies

It is consistent with results from the post-second cycle meta-evaluation, which showed that medical school professors and staff involved in accreditation preparation acknowledged its importance in identifying and addressing institutional weaknesses [12].

However, this study highlighted several challenges, particularly for medical schools preparing for accreditation. These institutions encountered resource constraints during the data collection, analysis, and reporting phases, which are integral to meeting the established criteria and procedures [13,14]. Although the significance of accreditation in enhancing quality is widely acknowledged, there was a marked discrepancy in perceptions between the site visit committee members and the administrative staff. Addressing the divergence in viewpoints between evaluators and medical schools is an urgent issue that demands immediate attention to facilitate a smooth accreditation preparation process. Considering the critical role that administrative staff play in this preparation, it is essential to improve their working conditions to allow them to focus effectively on their duties. Furthermore, it is worth considering offering compensation for their significant contributions to the accreditation process.

The study also revealed a general skepticism among both site visit committee members and self-evaluation study committee members toward the essential and excellence criteria of accreditation, possibly due to a lack of clear understanding and guidance. Although the overall impact of accreditation on improving the quality of medical education was viewed positively, the responses concerning the basic and excellence criteria from self-evaluation study committee members and site visit evaluators were less favorable than those from the post-2nd cycle meta-evaluation.

Nevertheless, the self-evaluation study committee members noted a significant shift in their perception of the site visit evaluators’ professionalism, with a more positive outlook in this study than in the post-2nd cycle evaluation. Similarly, the accuracy and reliability of the reporting process were viewed more favorably.

To streamline and strengthen the medical education accreditation process, it is essential to broaden support by providing additional technical, financial, and human resources, thereby alleviating the burden on faculty members. The KIMEE should provide clear and detailed guidelines for accreditation to facilitate systematic preparation. Regular training sessions are important to improve stakeholders’ understanding and preparedness for the accreditation process. Promoting better collaboration and communication between medical schools and accreditation bodies is vital to align their goals. Introducing incentives can mitigate the perceived burden of accreditation, increase motivation, and foster a positive outlook [15-17]. Providing consistent feedback and continually refining standards and procedures are crucial to enhancing the effectiveness of the process. Finally, adopting a data-driven approach for ongoing meta-evaluation of the process will enable informed, evidence-based improvements.

The meta-evaluation reveals critical implications for Korea’s medical education accreditation for enhanced stakeholder communication and collaboration, simplification of accreditation procedures, and improved support for administrative staff. Addressing skepticism with more explicit guidelines and training is critical for a positive accreditation perception. These steps are vital for fostering a transparent, efficient accreditation process that uplifts medical education standards in Korea.

### Limitations and generalizability

Using surveys can result in response bias because stakeholders may offer socially desirable responses or withhold their genuine opinions due to discomfort with the survey process. Furthermore, gathering data at a single time point can overlook the dynamic nature of perceptions, particularly when accreditation standards change. Reliance on self-reported surveys may also lead to bias, as participants might need to accurately report their behaviors or perceptions.

### Suggestions

This study emphasizes the need for longitudinal research to understand the long-term effects of medical school accreditation on medical education quality and outcomes. It also points out the significance of analyzing resource allocation strategies to improve the efficiency of the accreditation process. It calls for an examination of the clarity and suitability of accreditation standards. Finally, the study stresses the importance of fostering an environment and culture that prioritizes continuous quality improvement in medical education by incorporating lessons learned from the accreditation process into continuous professional development.

### Conclusion

The study sheds light on various stakeholders’ multifaceted perceptions of accreditation in medical education. Although there is a broadly positive attitude toward the essential nature and benefits of accreditation, differing opinions on the evaluation process, criteria, and the accrediting organization indicate a necessity for improved communication, the harmonization of expectations, and ongoing refinement of accreditation methods. These results highlight the significance of a cooperative approach to accreditation, one in which input from all parties is considered and integrated, fostering a transparent, equitable, and efficient process that ultimately elevates the standard of medical education.

## Figures and Tables

**Figure f1-jeehp-21-08:**
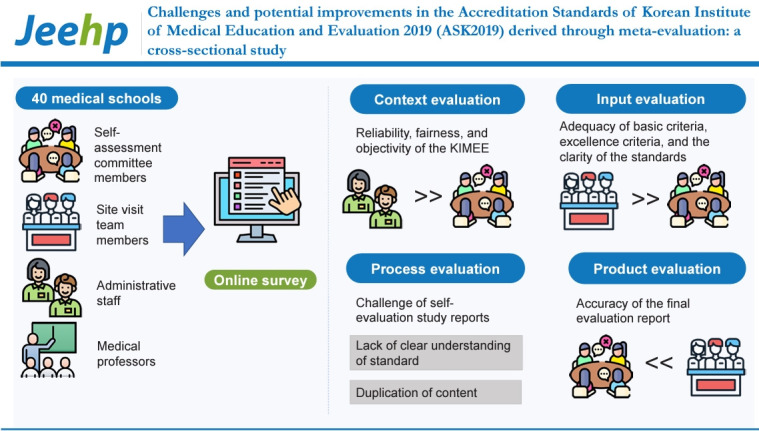


**Table 1.** Characteristics of participants

Region	Self-evaluation study committee members	Site visit committee members	Administrative staff	Medical school professors
Seoul	29 (16.9)	7 (25.0)	11 (15.9)	10 (15.9)
Busan	39 (22.7)	3 (10.7)	13 (18.8)	11 (17.5)
Daegu	20 (11.6)	4 (14.3)	5 (7.2)	4 (6.3)
Incheon	4 (2.3)	1 (3.6)	2 (2.9)	5 (7.9)
Daejeon	7 (4.1)	3 (10.7)	4 (5.8)	8 (12.7)
Gwangju	6 (3.5)	-	1 (1.4)	-
Gyeonggi-do	5 (2.9)	2 (7.1)	12 (17.4)	4 (6.3)
Gangwon-do	13 (7.6)	4 (14.3)	4 (5.8)	7 (11.1)
Chungcheongbuk-do	5 (2.9)	1 (3.6)	3 (4.3)	8 (12.7)
Chungcheongnam-do	2 (1.2)	1 (3.6)	4 (5.8)	-
Jeollabuk-do	22 (12.8)	1 (3.6)	1 (1.4)	1 (1.6)
Jeollanam-do	15 (8.7)	-	5 (7.2)	5 (7.9)
Gyeongsangbuk-do	1 (0.6)	-	1 (1.4)	-
Gyeongsangnam-do	3 (1.7)	-	2 (2.9)	-
Jeju-do	1 (0.6)	1 (3.6)	1 (1.4)	-
Total	172 (100.0)	28 (100.0)	69 (100.0)	63 (100.0)

Values are presented as number (%).

**Table 2.** Comparison of the necessity of accreditation for quality improvement in medical education according to the category of stakeholders

Variable	Mean±SD	F/P-value	Post hoc analysis
The necessity of accreditation for quality improvement in medical education		9.096/0.00^**^	B>A, C, D (Dunnett T3)
Self-evaluation study committee members (A)	4.56±0.80		
Site visit committee members (B)	5.43±0.84		
Administrative staff (C)	4.81±0.88		
Medical school professors (D)	4.48±1.12		

SD, standard deviation.

** P<0.01.

**Table 3.** Comparison between stakeholder categories regarding the degree of contribution to the development of medical education by accreditation and evaluation

Variable	Mean±SD	t-value	P-value
Degree of contribution to the development of medical education by accreditation and evaluation		1.30	0.19
Self-evaluation study committee members	4.42±0.85		
Medical school professors	4.26±0.98		

SD, standard deviation.

**Table 4.** Positive outcomes of medical school accreditation according to the self-evaluation study committee members and administrative staff

Variable	Positive outcomes of medical school accreditation				χ^2^
	Improvement of areas identified as deficient during self-evaluation study	Increased internal stakeholders’ interest	Establishment of an accreditation system for continuous quality improvement	Awareness and shared importance of societal accountability	
Group					0.82
Self-evaluation study committee members	132 (35.2)	98 (26.1)	118 (31.5)	27 (7.2)	
Administrative staff	49 (37.7)	29 (22.3)	43 (33.1)	9 (6.9)	
Sum	181 (35.8)	127 (25.1)	161 (31.9)	36 (7.1)	

Values are presented as number (%).

**Table 5.** Stakeholders’ perceptions of the reliability, fairness, and objectivity of the Korean Institute of Medical Education and Evaluation

Variable	Mean±SD	t-value	P-value
Reliability		-3.15	0.00^**^
Self-evaluation study committee members	4.31±0.93		
Administrative staff	4.68±0.74		
Fairness		-2.06	0.04^*^
Self-evaluation study committee members	4.26±0.93		
Administrative staff	4.52±0.76		
Objectivity		-4.68	0.00^**^
Self-evaluation study committee members	4.01±1.10		
Administrative staff	4.59±0.71		

SD, standard deviation.

*P<0.05. **P<0.01.

**Table 6.** Appropriateness and clarity of the evaluation and accreditation standards as perceived by stakeholders

Variable	Mean±SD	t-value	P-value
Adequacy of basic criteria		-2.52	0.01^*^
Self-evaluation study committee members	3.78±1.00		
Site visit committee members	4.28±0.90		
Adequacy of excellence criteria		-2.01	0.04^*^
Self-evaluation study committee members	3.08±1.21		
Site visit committee members	3.57±1.17		
Clarity of the standards		-2.02	0.04^*^
Self-evaluation study committee members	3.29±1.01		
Site visit committee members	3.71±1.12		

SD, standard deviation.

* P<0.05.

**Table 7.** Feasibility of implementing evaluation and accreditation standards in medical education as perceived by stakeholders

Variable	Mean±SD	F/P-value	Post hoc analysis
Feasibility of implementing accreditation standards in medical education		25.85/0.00^**^	B>A, C>A (Dunnett T3)
Self-evaluation study committee members (A)	3.43±0.97		
Site visit committee members (B)	4.11±0.92		
Administrative staff (C)	4.35±0.74		

SD, standard deviation.

** P<0.01.

**Table 8.** Comparison of the appropriateness of evaluation and accreditation procedures and methods as perceived by stakeholders

Variable	Mean±SD	t-value	P-value
Self-evaluation study evaluation		-2.97	0.00^**^
Self-evaluation study committee members	4.33±0.83		
Administrative staff	4.68±0.76		
Site visit		-2.59	0.01^*^
Self-evaluation study committee members	4.28±0.87		
Administrative staff	4.60±0.87		
Final evaluation report		-4.26	0.00^**^
Self-evaluation study committee members	4.30±0.86		
Administrative staff	4.71±0.58		
Interim evaluation report		-1.73	0.08
Self-evaluation study committee members	4.06±0.90		
Administrative staff	4.29±0.89		

SD, standard deviation.

*P<0.05. **P<0.01.

**Table 9.** Challenges encountered in the preparation of self-evaluation study reports

Variable	Challenges encountered in the preparation of self-evaluation study reports					χ^2^
	Lack of clear understanding of accreditation standards	Insufficient guidance for the preparation of self-evaluation study reports	Difficulty in writing duplication of content	Difficulty in qualitative assessment	Inadequate compensation for participating professors	
Group						34.71^***^
Self-evaluation study committee members	76 (23.8)	66 (20.7)	62 (19.4)	59 (18.5)	56 (17.6)	
Administrative staff	3 (2.4)	30 (24.4)	43 (35.0)	18 (14.6)	29 (23.6)	
Sum	79 (17.9)	96 (21.7)	105 (23.8)	77 (17.4)	85 (19.2)	

Values are presented as number (%).

*** P<0.001.

**Table 10.** Comparison between appropriateness of resource allocation to site visit evaluation

Variable	Mean±SD	t-value	P-value
Appropriateness of resource allocation		-3.23	0.00^**^
Self-evaluation study committee members	3.74±1.00		
Site visit committee members	4.39±0.99		
Cost appropriateness		0.49	0.63
Self-evaluation study committee members	3.73±0.95		
Site visit committee members	3.61±1.23		

SD, standard deviation.

** P<0.01.

**Table 11.** Comparison of perceptions of the professionalism of the site visit committee members

Variable	Mean±SD	t-value	P-value
Professionalism of the site visit committee members		-1.61	0.11
Self-evaluation study committee members	4.21±0.87		
Site visit committee members	4.50±1.00		

SD, standard deviation.

**Table 12.** Comparison of fidelity of the final evaluation report

Variable	Mean±SD	t-value	P-value
Accuracy of the final evaluation report		-3.31	0.00^**^
Self-evaluation study committee members	4.39±0.79		
Site visit committee members	4.89±0.74		

SD, standard deviation.

** P<0.01.

## Footnotes

**Data availability**
Data files are available from Harvard Dataverse: https://doi.org/10.7910/DVN/CQD7CX Dataset 1. Research data obtained from participants.

## Supplementary materials

Supplementary files are available from Harvard Dataverse: https://doi.org/10.7910/DVN/CQD7CX

Supplement 1. Survey questionnaires.

Supplement 2. Context, input, process, product evaluation criteria for ASK2019 program.

Supplement 3. Audio recording of the abstract.
