# Supplementary material for: Challenges and potential improvements in the Accreditation Standards of the Korean Institute of Medical Education and Evaluation 2019 (ASK2019) derived through meta-evaluation: a cross-sectional study
Source: J Educ Eval Health Prof. 2024 Apr 2;21:8. doi: 10.3352/jeehp.2024.21.8 (PMC11108703; doi:10.3352/jeehp.2024.21.8)
Supplement: Supplementary file 2 — Supplement 1. Survey questionnaires. [file jeehp-21-08-suppl1.docx]

**Supplementary 1.** Survey questionnaires

| **Components** | **Evaluation areas** | **Survey questions(6-Point Likert Scale)** |
| --- | --- | --- |
| Context Evaluations  : Overall perception of accreditation | Necessity of accreditation | How necessary do you believe accreditation is for quality improvement in medical education? |
|  | Contribution to accreditation and evaluation | To what degree do you think accreditation and evaluation contribute to the development of medical education? |
|  | Positive outcomes of accreditation | Select the Two Most Positive Outcomes of Medical School Accreditation:  -Improvement of areas identified as deficient during self-evaluation study  -Increased internal stakeholders’ interest  -Establishment of an accreditation system for continuous quality improvement  -Awareness and shared importance of societal accountability |
|  | Reliability, fairness, and objectivity of the KIMEE | How would you rate the degree of reliability in the operations and evaluations conducted by the Korean Institute of Medical Education and Evaluation (KIMEE) based on your personal experiences? |
|  |  | How would you rate the degree of fairness in the operations and evaluations conducted by the Korean Institute of Medical Education and Evaluation (KIMEE) based on your personal experiences? |
|  |  | How would you rate the degree of objectivity in the operations and evaluations conducted by the Korean Institute of Medical Education and Evaluation (KIMEE) based on your personal experiences? |
| Input Evaluation:  perception of accreditation standards | Appropriateness and clarity of accreditation standards | How adequate do you think the basic criteria are? |
|  |  | How adequate do you think the excellence criteria are? |
|  |  | How clear do you think the standards are in guiding medical education practices and accreditation processes? |
|  | Feasibility of implementing accreditation standards | How feasible do you find the implementation of accreditation standards in medical education by the Korean Institute of Medical Education and Evaluation (KIMEE)? |
| Process evaluation: perceptions of the accreditation process | Appropriateness of medical education accreditation procedures and methods | How appropriate do you find the evaluation of self-evaluation studies in the medical education accreditation process? |
|  |  | How appropriate do you find the evaluation presented in the site visit during the medical education accreditation process? |
|  |  | How appropriate do you find the evaluation presented in the final evaluation report during the medical education accreditation process? |
|  |  | How appropriate do you find the evaluation presented in the interim evaluation report during the medical education accreditation process? |
|  | Challenges encountered in the preparation of self-evaluation study reports | Please select the two options that you found most challenging during the preparation of the self-evaluation study report:  -Lack of clear understanding of accreditation standards  -Insufficient guidance for the preparation of self-evaluation study reports  -Difficulty in writing duplication of content  -Difficulty in qualitative assessment  -Inadequate compensation for participating professors |
| Product evaluation: perceptions of site visit evaluations | Appropriateness of resources allocated to site visits | How appropriate do you find the allocation of resources for site visit evaluations in the medical education accreditation process? |
|  |  | How appropriate do you find the allocation of cost for site visit evaluations in the medical education accreditation process? |
|  | Perceptions of the site visit committee members | How would you rate the professionalism of the site visit committee members? |
|  | Perceptions of site visit evaluation results | How accurate do you find the final evaluation report to be? |
