# Supplementary material for: Challenges and potential improvements in the Accreditation Standards of the Korean Institute of Medical Education and Evaluation 2019 (ASK2019) derived through meta-evaluation: a cross-sectional study
Source: J Educ Eval Health Prof. 2024 Apr 2;21:8. doi: 10.3352/jeehp.2024.21.8 (PMC11108703; doi:10.3352/jeehp.2024.21.8)
Supplement: Supplementary file 3 — Supplement 2. Context, input, process, product evaluation criteria for ASK2019 program. [file jeehp-21-08-suppl2.docx]

Supplement 1. Context, input, process, product evaluation criteria for ASK2019 program

| Components | Evaluation areas | Evaluation Criteria (Questions asked) |
| --- | --- | --- |
| Context Evaluations | Analyzing the needs of accredited medical schools | Has there been a sufficient analysis of the evaluated medical school's needs for accreditation? |
|  |  | Has there been an appropriate diagnosis of the current situation and realities for the medical school to undertake accreditation? |
|  | Setting goals for accreditation | Are the goals of the accreditation clearly presented? |
|  |  | Are the evaluated medical schools fully aware of the accreditation goals? |
|  | Analyzing the external environment for accreditation | Is there an appropriately established international collaborative system with foreign WFME-accredited accreditation organizations? |
|  |  | Do the stakeholders in accreditation process have appropriate interest, and is resistance being minimized? |
|  |  | Is there a clear legal and policy basis for accreditation and its evaluation? |
|  |  | Is there an appropriate analysis of the domestic and international educational, policy, and economic environment surrounding accreditation? |
|  | Analyzing the internal environment for accreditation | Is there sufficient financial capability to undertake accreditation? |
|  |  | Does the staff for overall planning and research of accreditation possess appropriate expertise? |
|  |  | Is the independence of the accreditation agency secured? |
| Input  Evaluations | Planning medical education accreditation | Is the accreditation validity period appropriate according to the results? |
|  |  | Is the duration of the accreditation process (from self-report submission to final judgment) appropriate? |
|  |  | Are the managing institution and organization suitable for operating accreditation? |
|  |  | Is the accreditation method appropriately set? |
|  |  | Are the accreditation procedures valid? |
|  |  | Is the purpose of accreditation valid? |
|  | Developing standards for medical education accreditation | Are the evaluation areas and items valid? |
|  |  | Are the accreditation standards appropriate for the context of domestic medical schools? |
|  |  | Is there a theoretical basis for the accreditation standards? |
|  |  | Are the accreditation standards sensitive to changes and demands? |
|  |  | Are there any standards that are not evaluated or difficult to evaluate? |
|  |  | Are the accreditation standards sufficient to achieve the evaluation's purpose? |
|  |  | Are the evaluation standards clearly presented in a way that promotes understanding? |
|  |  | Does the accreditation standard manual specify the accreditation and judgment criteria clearly and concretely? |
|  |  | Are the criteria for grading each evaluation standard clear? |
|  |  | Is the procedure for revising the accreditation system valid? |
|  |  | Is the timing of announcing and applying revised accreditation standards appropriate? |
|  | Human resources for accreditation standards development | Do the researchers involved in the development of accreditation standards possess professional competencies? |
|  |  | Is there a sufficient number of research personnel to carry out the development of accreditation standards? |
|  |  | Is the amount and composition of administrative personnel supporting evaluation personnel appropriate? |
|  | Accreditation briefing | Is the content of the briefing sufficient for understanding accreditation? (Qualitative adequacy of education) |
|  |  | Are the amount and frequency of briefings sufficient for understanding accreditation? (Quantitative adequacy of education) |
|  |  | Are the timing and location of the briefings appropriate? |
|  |  | Do the personnel responsible for the briefing have sufficient expertise? |
|  |  | Are adequate materials provided to understand the briefing? |
|  |  | Was there a smooth Q&A session during the briefing? |
|  | Self-evaluation study template and guidelines | Are the template and guidelines for writing the self-evaluation study report specifically presented? |
|  | Self-evaluation study report workshops | Is the content of the workshop sufficient to understand how to write a self-evaluation study report? (Qualitative adequacy of education) |
|  |  | Are the volume and frequency of the workshops sufficient for understanding accreditation? (Quantitative adequacy of education) |
|  |  | Are the timing and location of the workshops appropriate? |
|  |  | Do the personnel conducting the workshop training have appropriate expertise? |
|  |  | Are adequate materials provided to understand the workshop? |
|  |  | Was there a smooth Q&A session during the workshop? |
|  |  | Is it adequately checked whether the educational objectives are achieved after the workshop? |
|  | Evaluator pool selection | Is the evaluator pool comprised of members with professional competencies? |
|  |  | Is the size and composition of the evaluator pool appropriate? |
|  |  | Are the procedures and methods for selecting the evaluator pool appropriate? (Are the criteria for recommending evaluators from universities appropriate?) |
|  | Evaluator pool training | Is the content of the workshop sufficient to understand how to evaluate a self-evaluation study report? (Qualitative adequacy of education) |
|  |  | Are the volume and frequency of the workshops sufficient for understanding accreditation? (Quantitative adequacy of education) |
|  |  | Are the timing and location of the workshops appropriate? |
|  |  | Do the personnel conducting the workshop training have sufficient expertise? |
|  |  | Are adequate materials provided to understand the workshop? |
|  |  | Was there a smooth Q&A session during the workshop? |
|  |  | Is it adequately checked whether the evaluator pool has acquired the appropriate competencies after the training? |
|  | Evaluator selection | Is the evaluation committee (site evaluation team) composed of members with professional competencies? |
|  |  | Is the size and composition of the evaluation committee (site evaluation team) appropriate? |
|  |  | Are the procedures, methods, and criteria for selecting the evaluation committee (site evaluation team) appropriate? |
|  |  | Are appropriate evaluators assigned for each area? |
|  | Clarity of the evaluation guide | Is the evaluation guide (for written and site visit evaluations) clear enough to ensure fair and objective evaluations by the evaluators? |
|  | Evaluator training | Is the training content sufficient for understanding accreditation? (Qualitative adequacy of education) |
|  |  | Are the volume and frequency of the training sufficient for understanding accreditation? (Quantitative adequacy of education) |
|  |  | Are the timing and location of the training appropriate? |
|  |  | Do the personnel conducting the training have appropriate expertise? |
|  |  | Are adequate materials provided for understanding the training? |
|  |  | Was there a smooth Q&A session during the training? |
|  |  | Is it adequately checked whether the site visit team has acquired appropriate competencies after the training? |
| Process  Evaluations | Written assessment of the self-evaluation study report | Is the time period for evaluating the self-evaluation study report sufficient? (Is the timing of provision to evaluators appropriate?) |
|  |  | Are sufficient opportunities for communication provided among evaluators? |
|  |  | Is reliability secured among evaluators in evaluating the self-evaluation study report? |
|  |  | Are evaluators diligently conducting the written assessment of the self-evaluation report? |
|  | Site visit evaluation | Is the evaluation subject efficiently preparing for the site visit with clear pre-visit preparation and requirements communicated? |
|  |  | Are the timing and duration of the site visit appropriate? |
|  |  | Is the evaluation time appropriately allocated for each evaluation area? |
|  |  | Is the procedure for conducting the site visit appropriate? |
|  |  | Are the quantity and quality of the evaluation materials adequate for evaluation? |
|  |  | Did appropriate respondents from the evaluated medical school attend and respond during the site visit? |
|  |  | Is there smooth and sufficient communication between evaluators and respondents? |
|  |  | Is the site visit conducted ethically and with respect for the dignity of the evaluation subjects? |
|  |  | Does the site visit thoroughly verify the situation on-site? |
|  |  | Is the on-site evaluation conducted fairly among medical schools? |
|  | Final evaluation  process and reporting of final evaluation results | Is the derivation of evaluation results conducted in rational steps? |
|  |  | Is reliability secured among the evaluation panel? |
|  |  | Are fair decision-making opportunities provided to the evaluation chair and evaluators? |
|  |  | Is the evaluation result report comprehensive and systematic? |
|  | Improvement plan | Are the standards and format for writing an improvement plan clearly presented? |
|  |  | Are the timing and duration for writing the improvement plan appropriate? |
|  |  | Does the preparation of the improvement plan provide practical help in the improvement efforts of the accredited medical school? |
|  |  | Is it valid to mandatorily use the improvement plan for interim evaluations? |
|  | Interim evaluations | Are the evaluation standards for interim evaluations appropriate? |
|  |  | Are the timing and intervals of interim evaluations appropriate? |
|  |  | Are the methods and procedures for interim evaluations appropriate? |
|  | Response and coordination to objections | Are adequate opportunities provided for objections from the accredited medical school to the evaluation results? |
|  |  | Are the coordination and derivation procedures for the evaluators' responses to the objections rational? |
|  |  | Are the evaluators adequately responding to objections from the accredited university? |
| Product  Evaluations | Identification of areas for improvement | Are the directions for improvement based on the evaluation results valid and appropriately presented?? |
|  |  | Are the evaluation results specific and clear enough to aid in improving the accredited medical school's education? |
|  |  | Can the accredited medical school interpret the evaluation results without difficulty? |
|  |  | Are the evaluation results described based on credible evidence that can be accepted by the evaluation subjects and stakeholders? |
|  | Finalization and reporting of accreditation results | Is the evaluation judgment fairly assessed by medical schools? |
|  |  | Is the final evaluation report comprehensive and systematic? |
|  |  | Are the evaluation results delivered to the accredited medical schools on schedule? |
|  |  | Can the evaluation judgment be trusted? |
|  |  | Is the method, scope, and level of disclosing the evaluation results appropriate? |
|  | Impact of accreditation | Is the accreditation useful for improving and developing educational programs and activities of the medical school? |
|  |  | Are the evaluation results appropriately utilized in related policies, providing support or benefits to students, programs, and the medical school? |
|  |  | Does the evaluation contribute to increasing competitiveness in industry, society, and the medical field? |
|  | Improving accreditation | Is meta-evaluation being appropriately conducted to improve the quality of accreditation? |
|  |  | Are there sufficient continuous improvement efforts by the accreditation organization for high-quality evaluation? |

Supplement 2. Data collection methods for evaluation of the ASK2019 program

| Components | Evaluation areas | Data collection methods |
| --- | --- | --- |
| Context Evaluations | Analyzing the needs of accredited medical schools | Report on the evolution and future directions accreditation standards |
|  |  | Public hearing materials for amendments to accreditation standards |
|  |  | Research report to understand the current state of medical education in medical schools |
|  | Setting goals for accreditation | Objectives of the education accreditation organization |
|  |  | Surveys and interviews with self-evaluation study committee members |
|  | Analyzing the external environment for accreditation | Exchange contents and status with international organizations by medical domestic education accreditation organization |
|  |  | Surveys and interviews with self-evaluation study committee members, medical school faculty members, administrative staff. |
|  |  | Relevant laws and regulations |
|  |  | Research report to understand the current state of medical education in medical schools |
|  | Analyzing the internal environment for accreditation | Financial reports of medical education accreditation organization |
|  |  | Organizational chart and members of medical education accreditation organization |
|  |  | Career histories of planning and research personnel |
|  |  | Relevant laws and regulations |
|  |  | Surveys and interviews with site visit team members, secretariat staff. |
| Input  Evaluations | Planning medical education accreditation | Surveys and interviews with site visit team members, self-evaluation study committee members, secretariat staff |
|  |  | Materials related to the organization's expertise |
|  |  | Public materials of accreditation objectives |
|  | Developing standards for medical education accreditation | Medical education accreditation standards |
|  |  | Surveys and interviews with site visit team members, self-evaluation study committee members |
|  |  | Research report to understand the current state of medical education in medical schools |
|  |  | Report on the evolution and significance of medical education accreditation standards |
|  |  | Examples of Q&A about accreditation standards |
|  |  | Objectives of accreditation organization’s evaluation project |
|  |  | Accreditation standards committee materials |
|  |  | Meeting records of standard establishment and revision committees |
|  | Human resources for accreditation standards development | Selection and competency verification materials for researchers involved in accreditation standards development |
|  |  | Staffing for reviewing materials in relation to the workload of standard development research |
|  |  | Size and composition materials of administrative staff supporting evaluation personnel |
|  | Accreditation briefing | Materials of accreditation briefing |
|  |  | Materials for evaluating the satisfaction of participants with the accreditation briefing |
|  |  | Timing and location status materials of the briefing |
|  |  | Current status of personnel responsible for briefing education |
|  |  | Surveys and interviews with self-evaluation study committee members |
|  | Self-evaluation study template and guidelines | Self-evaluation report guideline materials |
|  |  | Surveys and interviews with self-evaluation study committee members |
|  | Self-evaluation study report workshops | Materials for self-evaluation study report workshops |
|  |  | Materials on the volume and frequency of workshops |
|  |  | Materials on the timing and location of workshops |
|  |  | Current status of personnel responsible for workshop education |
|  |  | Materials for evaluating satisfaction with the workshop |
|  |  | Workshop summary materials (Q&A) |
|  |  | Surveys and interviews with self-evaluation study committee members |
|  | Evaluator pool selection | History materials of the evaluator pool |
|  |  | Size and composition materials of evaluator pool |
|  |  | Criteria, procedures, and methods materials for selecting evaluator pool |
|  |  | Request letters for evaluator pool selection |
|  | Evaluator pool training | Materials on the history of the evaluator pool |
|  |  | Materials on the volume and frequency of workshops |
|  |  | Materials on the timing and location of workshops |
|  |  | Current status of personnel responsible for workshop education |
|  |  | Materials for evaluating satisfaction with the workshop |
|  |  | Workshop summary materials (Q&A) |
|  |  | Surveys and interviews with site visit team members |
|  | Evaluator selection | Materials on the history of the evaluator committee |
|  |  | Materials on the size and composition of the evaluator committee |
|  |  | Criteria, procedures, and methods materials for evaluator selection |
|  |  | Request letters for evaluator selection |
|  |  | Basis for evaluator assignment |
|  | Clarity of the evaluation guide | Materials for the evaluation guide |
|  |  | Evaluator committee guide review materials |
|  | Evaluator training | Review materials of evaluator training content |
|  |  | Materials for evaluating satisfaction with the evaluator committee |
|  |  | Materials on the timing and location of evaluator committee education |
|  |  | Current status of personnel responsible for evaluator education |
|  |  | Materials for evaluating satisfaction with evaluator training |
|  |  | Surveys and interviews with site visit team members, self-evaluation study committee members |
| Process  Evaluations | Written assessment of self-evaluation study report | Evaluation timing and schedule data for the written evaluation |
|  |  | Surveys and interviews with site visit team members, self-evaluation study committee members |
|  |  | Meeting minutes of self-evaluation report evaluation committee |
|  |  | Judgment committee meeting minutes |
|  |  | Self-evaluation study reports |
|  |  | Objection records according to self-evaluation study reports written assessment |
|  | Site visit evaluation | Official letters sent to accredited medical schools |
|  |  | Data on the timing and duration of site visit evaluations |
|  |  | Surveys and interviews with site visit team members, secretariat staff, self-evaluation study committee members, medical students |
|  |  | Data on the evaluation time for each area |
|  |  | Data on the procedures and adequacy review for site visits |
|  |  | Site verification status data |
|  |  | Review data for essential evaluation materials |
|  |  | Meeting minutes of the site visit team |
|  |  | Objection records according to evaluation reports |
|  |  | Judgment committee meeting minutes |
|  | Final evaluation  process and reporting of final evaluation results | Stage-specific status data for deriving evaluation results |
|  |  | Meeting minutes of the evaluator committee |
|  |  | Judgment committee meeting minutes |
|  |  | Surveys and interviews with site visit team members |
|  |  | Consent data of the evaluation panel on evaluation results |
|  |  | Consent data on fair participation of evaluators |
|  |  | Evaluation result reports |
|  | Improvement plan | Request letters for writing improvement plans |
|  |  | Surveys and interviews with accredited medical school faculty members, self-evaluation study committee members |
|  | Interim evaluations | Methods, timing, and interval status for interim evaluation |
|  |  | Self-review data of the evaluation committee |
|  |  | Surveys and interviews with self-evaluation study committee members, site visit team members |
|  | Response and coordination to objections | Surveys and interviews with self-evaluation study committee members |
|  |  | Judgment committee meeting minutes |
| Product  Evaluations | Identification of areas for improvement | Final result reports |
|  |  | Surveys and interviews with accredited medical school faculty and staff |
|  |  | Evaluation result evidence |
|  | Finalization and reporting of accreditation results | Judgment committee judgment progression data |
|  |  | Final commentary |
|  |  | Final result delivery schedule |
|  |  | Final result reports |
|  |  | Site visit evaluation reports |
|  |  | Judgment committee meeting minutes |
|  |  | Written evaluation reports |
|  |  | External disclosure of accreditation results |
|  | Impact of accreditation | Pre-and post-accreditation comparison research reports |
|  |  | Utilization status of evaluation results |
|  | Improving accreditation | Meta-evaluation performance reports |
|  |  | Improvement/long-term/short-term development plan reports of the evaluation organization |

Supplement 3. Summary of stakeholder survey results

A total of 331 individuals participated in the input evaluation process, including 172 self-evaluation committee members, 27 site visit committee members, 69 administrative staff, and 63 medical school professors.

| Evaluation Criteria (Questions asked) | Group of participants | 6-point Likert scale^1)^ | SD^2)^ |
| --- | --- | --- | --- |
| Are the evaluated medical schools fully aware of the accreditation goals? | Self-evaluation committee members | 4.56 | 0.80 |
|  | Site visit committee members | 5.43 | 0.84 |
|  | Administrative staff | 4.81 | 0.88 |
|  | Medical school professors | 4.48 | 1.12 |
| Are the accreditation standards appropriate for the context of domestic medical schools? | Self-evaluation committee members | 3.43 | 0.97 |
|  | Site visit committee members | 4.11 | 0.92 |
|  | Administrative staff | 4.35 | 0.74 |
| Is there sufficient financial capability to undertake accreditation? (Appropriateness of resources allocation) | Self-evaluation committee members | 3.74 | 1.00 |
|  | Site visit committee members | 4.39 | 0.99 |
| Is there sufficient financial capability to undertake accreditation? (Cost appropriateness) | Self-evaluation committee members | 3.73 | 0.95 |
|  | Site visit committee members | 3.61 | 1.23 |
| Are the evaluation areas and items valid? (Korea basic standards) | Self-evaluation committee members | 3.78 | 1.00 |
|  | Site visit committee members | 4.28 | 0.90 |
| Are the evaluation areas and items valid? (high quality development standards) | Self-evaluation committee members | 3.08 | 1.21 |
|  | Site visit committee members | 3.57 | 1.17 |
| Are the evaluation standards clearly presented in a way that promotes understanding? | Self-evaluation committee members | 3.29 | 1.01 |
|  | Site visit committee members | 3.71 | 1.12 |
| Can the evaluation judgment be trusted? | Self-evaluation committee members | 4.31 | 0.93 |
|  | Administrative staff | 4.68 | 0.74 |
| Is the evaluation judgment fairly assessed by medical schools? | Self-evaluation committee members | 4.26 | 0.93 |
|  | Administrative staff | 4.52 | 0.76 |
| Are the evaluation results described based on credible evidence that can be accepted by the evaluation subjects and stakeholders? | Self-evaluation committee members | 4.01 | 1.10 |
|  | Administrative staff | 4.59 | 0.71 |
| Are evaluators diligently conducting the written assessment of the self-evaluation report? | Self-evaluation committee members | 4.33 | 0.83 |
|  | Site visit committee members | 5.00 | 0.77 |
|  | Administrative staff | 4.68 | 0.76 |
| Is the procedure for conducting the site visit appropriate? | Self-evaluation committee members | 4.28 | 0.87 |
|  | Site visit committee members | 5.00 | 0.77 |
|  | Administrative staff | 4.60 | 0.87 |
| Is the derivation of evaluation results conducted in rational steps? | Self-evaluation committee members | 4.30 | 0.86 |
|  | Site visit committee members | 5.00 | 0.77 |
|  | Administrative staff | 4.71 | 0.58 |
| Are the methods and procedures for interim evaluations appropriate? | Self-evaluation committee members | 4.06 | 0.90 |
|  | Site visit committee members | 5.00 | 0.77 |
|  | Administrative staff | 4.29 | 0.89 |
| Is the site visit conducted ethically and with respect for the dignity of the evaluation subjects? | Self-evaluation committee members | 4.21 | 0.87 |
|  | Site visit committee members | 4.50 | 1.00 |
| Is the final evaluation report comprehensive and systematic? | Self-evaluation committee members | 4.39 | 0.79 |
|  | Site visit committee members | 4.89 | 0.74 |
| Is the accreditation useful for improving and developing educational programs and activities of the medical school? | Self-evaluation committee members | 4.42 | 0.85 |
|  | Medical school professors | 4.26 | 0.98 |

1) 5-point Likert scale: 1 = Strongly Disagree, 6 = Strongly Agree

2) SD: Standard Deviation

| Evaluation Criteria (Questions asked) | Variables | Group of participants | No. (%) |
| --- | --- | --- | --- |
| Challenges encountered in the preparation of self-evaluation study reports | Lack of clear understanding of accreditation standards | Self-evaluation committee members | 76(23.8) |
|  |  | Administrative staff | 3(2.4) |
|  | Insufficient guidance for the preparation of self-evaluation study reports | Self-evaluation committee members | 66(20.7) |
|  |  | Administrative staff | 30(24.4) |
|  | Difficulty in writing duplication of content | Self-evaluation committee members | 62(19.4) 59(18.5) |
|  |  | Administrative staff | 43(35.0) |
|  | Difficulty in qualitative assessment | Self-evaluation committee members | 59(18.5) |
|  |  | Administrative staff | 18(14.6) |
|  | Inadequate compensation for participating professors | Self-evaluation committee members | 56(17.6) |
|  |  | Administrative staff | 29(23.6) |
| Are the evaluation results appropriately utilized in related policies, providing support or benefits to students, programs, and the medical school? | Improvement of areas identified as deficient during self-evaluation study | Self-evaluation committee members | 132(35.2) |
|  |  | Administrative staff | 49(37.7) |
|  | Increased internal stakeholders' interest | Self-evaluation committee members | 98(26.1) |
|  |  | Administrative staff | 29(22.3) |
|  | Establishment of accreditation system for continuous quality improvement | Self-evaluation committee members | 118(31.5) |
|  |  | Administrative staff | 43(33.1) |
|  | Awareness and shared importance of societal accountability | Self-evaluation committee members | 27(7.2) |
|  |  | Administrative staff | 9(6.9) |

Reference: Lee SH, Kang SH, Kang YJ, Kang MR, Lee WY, Lim MS, et al. Meta evaluation study for ASK2019 (Accreditation Standards of KIMEE 2019) Accreditation. Seoul: KIMEE; 2023 Oct 10.

Supplement 4. Summary of stakeholder focus group

The study included 40 self-evaluation committee members across 14 focus group interviews, 7 site visit committee members in 2 interviews, and both secretariat staff participated in 1 interview, with 3 participants from each group.

| Interview Subject | Major Theme | Subtheme |
| --- | --- | --- |
| Self-evaluation committee members | A. General perception of accreditation | (1) purpose of evaluation and accreditation: process for continuous quality improvement (CQI) of medical schools  (2) opportunity for work cooperation: using accreditation as a pretext to encourage participation in educational tasks |
|  | B. Characteristics of the self-evaluation study committee composition | (1) committee recruitment: difficulty in securing sufficient resources  (2) continuous participation: self-evaluation study committee members, 'an inescapable rut'  (3) understanding of standards: variance among universities depending on the presence of medical education experts  (4) university support: the scale of support varies depending on the enthusiasm of the headquarters and foundation  (5) administrative staff: 'full participation' hindered by contract conditions and employment types  (6) need for staff training: varying recognition of the importance of administrative support by university |
|  | C. Characteristics in the preparation process for accreditation | (1) understanding accreditation standards: difficult and ambiguous to comprehend  (2) setting goals for accreditation results: 'let's not just get it for 2 years vs. Let's definitely get it for 6 years'  (3) site visit team: the attitude and evaluation expertise of the evaluators is important  (4) consulting opinions: whether it's a 'suggestion' or a 'mandatory requirement'  (5) preparation of materials: realization of appendices and available documents  (6) interviews with faculty and students: doubts about the effectiveness of interview results |
|  | D. Perception of interim evaluation | (1) objective of interim evaluation: opportunity for improvement before the main evaluation  (2) sense of burden from evaluation: feels like a second main evaluation  (3) report preparation: focused on writing about improvements  (4) accreditation results: feedback that feels insincere |
|  | E. Perception of judgment results and improvement plans | (1) judgment results: 'why a good evaluation can be poisonous'  (2) evaluation results: need to improve the grading system evaluation judgment such as '2, 4, 6 years'  (3) improvement plans: 'promises that cannot be kept'  (4) appeals: self-inflicted position of being 'the evaluated' |
| Site visit committee members | A. Selection of site visit committee members and composition of the evaluation team | (1) selection of members considering medical education experience and expertise  (2) site visit team workshop: recognition of the importance of pre-training |
|  | B. Learning and limitations in site visit activities | (1) learning experiences through participation in accreditation activities  (2) smooth and equitable communication structure within the evaluation team |
|  | C. Efforts to restore trust in evaluation and accreditation | (1) phased review to enhance the reliability of accreditation results  (2) efforts to form a reciprocal relationship between evaluators and evaluated universities |
| Secretariat staff | A. Related to evaluation and accreditation work | (1) coordination of schedules related to site visits  (2) communication with evaluated universities and site visit teams  (3) points for improvement to enhance expertise in accreditation |
|  | B. Related to secretariat operation | (1) human resources and infrastructure  (2) staff competencies and roles |

Reference: Lee SH, Kang SH, Kang YJ, Kang MR, Lee WY, Lim MS, et al. Meta evaluation study for ASK2019 (Accreditation Standards of KIMEE 2019) Accreditation. Seoul: KIMEE; 2023 Oct 10.
